# Older Adult Care Career Intention and Associated Factors Among Higher Vocational College Students: A Cross‐Sectional Study

**DOI:** 10.1002/nop2.70578

**Published:** 2026-05-18

**Authors:** Baohua Liu, Yuchen Ying, Nan Sun, Laiyou Li, Feng Wang, Zhenzhen Xu, Hongguo Zhou, Shuping Zhou

**Affiliations:** ^1^ Ningbo College of Health Sciences, School of Health Services and Wellness Zhejiang China

**Keywords:** aging attitudes, contact, Fraboni scale of ageism, geriatric career intention, older adult care, workforce

## Abstract

**Aim:**

The aim of this study was to describe the career intention and identify its associated factors among higher vocational college students majoring in older adult care‐related fields in China.

**Design:**

A cross‐sectional study.

**Methods:**

The survey was conducted between May and July 2025 using a self‐report questionnaire, with a total of 352 valid samples ultimately included. The outcome variable was assessed with a dichotomous (willing/unwilling) item regarding willingness to pursue a career in older adult care after graduation. Descriptive analysis and hierarchical logistic regression were performed to examine the associations between career intention and potential associated factors.

**Results:**

Approximately half of the participants indicated a willingness to pursue a career in older adult care work. The final optimal model (Model 3) of hierarchical logistic regression revealed that male gender (OR = 2.135, 95% CI = 1.022–4.776), majoring in aged health care and management (OR = 3.138, 95% CI = 1.505–6.544), living with older adults (OR = 1.639, 95% CI = 1.014–2.650), and higher frequency of volunteering for older adults (≥ 3 times) (OR = 1.945, 95% CI = 1.092–3.465) were positively associated with career intention in OAC. Meanwhile, ageism was negatively associated with the intention to pursue a career in OAC (OR = 0.972, 95% CI = 0.947–0.997).

**Conclusion:**

This study suggests that students' majors, interactions with older adults, and ageism are associated with their future career intention in OAC. The factors identified can be used to guide the development of talent cultivation strategies for OAC in order to promote the development of OAC human resources in China.

**Impact:**

It is essential to incorporate intergenerational interaction opportunities into the curricula and practical training of students preparing for careers in older adult care in China.

**Reporting Method:**

This study followed the STROBE guidelines for cross‐sectional research.

**Patient or Public Contribution:**

This study did not involve patients or the public in its design, conduct or reporting.

**Abbreviations**
CI: confidence interval
FSA: Fraboni Scale of Ageism
KAOP: Kogan's Attitudes toward Older People Scale
OAC: older adult care
OR: odds ratio
SD: standard deviation
VIF: variance inflation factors

## 1 Introduction

Corresponding to the increase in life expectancy, the aged population gradually experiences functional decline, which leads to a decrease in their ability to live independently (Singh‐Manoux et al. 2012; Krall et al. 2014). Coupled with the high prevalence of chronic diseases and geriatric syndromes, these factors lead to complex and multifaceted care needs (Chen et al. 2022, 2023). The traditional model in which younger generations care for their older ones is no longer sustainable. Accordingly, there is a global recognition of the need for well‐trained professionals to meet these complex needs (Kiljunen et al. 2019; Watson et al. 2020). However, this surging demand for care services stands in stark contrast to a critical shortage of skilled long‐term care workforce. More importantly, there is a widespread lack of interest among young professionals in pursuing careers in the older adult care (OAC) sector (Koskinen et al. 2022; Dai et al. 2021).

In China, the development of human resources for OAC is hindered by staffing shortages, high turnover, low remuneration and negative social perceptions of care workers (Qi et al. 2024). Most OAC providers are middle‐aged women with limited education and inadequate professional training (Qi et al. 2024; Song et al. 2014). To strengthen the OAC workforce, 12 national departments jointly issued the policy document ‘Opinions on Strengthening the Construction of the Older adult Care Service Talent Team’ in 2024. The document emphasizes the need to strengthen the development of nursing, aged health care, and management and other related majors in vocational education to meet the huge needs of OAC human resources (Ministry of Civil Affairs of the People's Republic of China 2023). Aged health care and management is a new vocational education programme established in 2016 to address the care needs of China's aging population. It aims to cultivate professionals capable of delivering comprehensive services for older adults, including basic daily care, health management, psychological support, recreational activities, and geriatric social work. Unlike traditional nursing programmes, which focus primarily on clinical care, this major emphasizes service provision in non‐hospital settings.

Working with older adults is an unpopular career choice among students majoring in healthcare professions (such as clinical medicine, nursing, and rehabilitation) (Hebditch et al. 2020). Their career intention to work with older adults is further shaped by factors such as gender, years of study, experience of contact with older adults, attitudes toward older adults, and ageism (Zhao et al. 2020; Vitman‐Schorr and Rozani 2025; Wang et al. 2024; Guo et al. 2021). However, few studies focused on the career intentions among higher vocational education students majoring in OAC programmes.

Given the severe shortage of OAC personnel, there is an urgency to study the career intentions and associated factors of higher vocational college students in nursing and other OAC‐related majors to inform the relevant authorities on the direction of these students, which will impact the climate of the Chinese OAC.

## 2 Methods

### 2.1 Ethics Approval and Consent to Participate

This study was approved on April 2023 by the Ethics Committee of Ningbo College of Health Sciences (approval number: 2023‐006). All participants were informed about the goal of this study. Data were collected anonymously. All methods were carried out in accordance with the Declaration of Helsinki.

### 2.2 Study Design and Sample Size

This research employed a cross‐sectional, questionnaire‐based, descriptive survey design. The survey was carried out from May to July 2025 at the affiliated institution of the researchers, utilizing a convenience sampling approach. The questionnaire was distributed in class among students from two majors related to OAC, namely nursing and aged health care and management. The participants included first and second‐year students majoring in nursing (3‐year programme, *n* = 277) and aged health care and management (3‐year programme, *n* = 147). The exclusion criterion was incomplete response to the questionnaire. The questionnaires were submitted anonymously. All participants were informed of the research objectives and signed informed consent forms. All students were assured that their responses would be kept confidential.

### 2.3 Study Variables

#### 2.3.1 Willingness to Pursue a Career in OAC (Career Intention)

The dependent variable was assessed using a single dichotomous item adapted from prior research (Zhao et al. 2020; Racic et al. 2019; Carlson and Idvall 2015; Shen and Xiao 2012): ‘Are you willing to pursue an older adult care related career after graduation?’ (1 = willing, 0 = unwilling).

#### 2.3.2 Sociodemographic and Geriatric‐Related Characteristics

The demographic characteristics of the participants included age, gender (1 = Male, 0 = Female), major (1 = Aged health care and management, 0 = Nursing) and academic year (1 = First year, 0 = Second year). Participants' older adult related factors included whether they lived with older adults (≥ 60 years old) when returning home (1 = Yes, 0 = No), whether they had taken courses in geriatric physiology (1 = Yes, 0 = No), and geriatric psychology (1 = Yes, 0 = No), and the frequency of volunteer services for the older adults (1 = ≥ 3 times, 0 = < 3 times). Additionally, participants were inquired about their preferred client age groups for service provision, which were classified as follows: infants (0–1 years), children (2–12 years), adolescents (13–18 years), young adults (19–39 years), middle‐aged adults (40–59 years) and older adults (≥ 60 years).

### 2.4 Fraboni Scale of Ageism (FSA)

The Fraboni scale of ageism is a reliable and valid scale for measuring the degree of discrimination against older adults (Fraboni et al. 1990). The measurement consists of 29 items: 10 for anti‐locution, nine for discrimination, and 10 for avoidance. It is rated on a 4‐point Likert scale ranging from 1 (strongly disagree) to 4 (strongly agree). The total score ranges from 29 to 116; the higher the total score, the greater the ageism toward older adults.

### 2.5 Kogan's Attitudes Toward Older People Scale (KAOP)

The Kogan attitudes toward older adults scale was used to measure students' attitudes toward older adults (Kogan 1961). The KAOP is a 34‐item Likert‐type scale, with half negatively worded and half positively worded, and has been used extensively to measure attitudes toward older people (Hammad et al. 2025; González‐Moreno et al. 2025). The KAOP in our study was scored on a 6‐point Likert scale ranging from 6 (highly positive) to 1 (highly negative). The total score range was 34–204; a higher KAOP score indicated a more positive attitude. Kogan investigated the scale's reliability and reported Spearman–Brown reliability coefficients ranging from 0.66 to 0.83.

### 2.6 Statistical Analysis

All analyses were performed using SPSS 24.0. Skewness, kurtosis tests and the Q–Q plot were used to examine the normality of the distribution for each continuous data set (age, FSA and KAOP). According to Kim, for samples > 300, an approximate normal distribution was defined for variables with absolute values of skewness below 3 and kurtosis below 8 (Kim 2013). The results showed that the skewness values of each continuous variable ranged from −0.45 to 0.83, and the kurtosis values of each continuous variable ranged from 0.16 to 1.62, which is indicative of a normal distribution (Kim 2013). The Q–Q plot results indicated that the variables of age, FSA and KAOP did not deviate significantly from a normal distribution.

Descriptive statistics were analyzed using frequencies and percentages for binary variables, and means and standard deviations (SD) were calculated for continuous variables. The correlations between continuous variables were analyzed using Pearson correlation analysis. Chi‐square and *t*‐tests were employed to compare independent variables between the group willing to pursue a career in OAC and the group unwilling to pursue a career in OA**C**. Chi‐square tests were used for categorical variables, and independent *t*‐tests for continuous variables. All variables were included in the multivariable analysis.

Hierarchical logistic regression models were used to test the impact of variables on career intention (dependent variable). Hierarchical logistic regression was conducted with four models. Model 1 incorporated sociodemographic variables (age, gender, grade, major, course, living with older adult, and volunteer services) and established a baseline; Model 2 added KAOP to Model 1; Model 3 added FSA to Model 1; Model 4 added both FSA and KAOP to Model 1. The following assumptions for logistic regression were assessed. First, multicollinearity among independent variables was evaluated using variance inflation factors (VIF); VIF values ranged from 1.07 to 3.60, indicating no serious multicollinearity. Second, the linearity of continuous variables (age, FSA, and KAOP) with respect to the logit of the dependent variable was examined; no violations were detected (*p* > 0.05). The Hosmer–Lemeshow tests were used to evaluate the goodness of fit for each of the three logistic regression models, and a *p* > 0.05 for each test indicated acceptable model fit. Odds ratios (OR) and 95% confidence intervals (CI) for OR were calculated.

The test level *α* = 0.05, and *p* < 0.05 was considered statistically significant.

## 3 Results

### 3.1 Participants' Characteristics

In total, 352 students completed the questionnaires, and all questionnaires were fully completed; thus, all participants (100%) were included in the final analysis, yielding an overall response rate of 83.0% (352/424). The response rates for nursing and aged health care and management were 78.7% (218/277) and 91.2% (134/147), respectively.

In this study, FSA and KAOP showed satisfactory internal consistency, with a Cronbach's alpha coefficient of 0.87 for both scales.

As shown in Table 1, the average age was 19.75 ± 1.05 years. Most participants were women (86.1%), and 50.3% were first‐year students. Participants' majors included nursing (61.9%) and aged health care and management (38.1%). Mean scores were 64.7 (SD = 9.45) for FSA and 143.2 (SD = 14.17) for KAOP.

**TABLE 1:** Participant characteristics (*N* = 352).

Characteristics	*n* (%)	Mean ± SD
Age	—	19.75 ± 1.05
Gender		
Male	49 (13.9)	—
Female	303 (86.1)	
Major		
Aged health care and management	134 (38.1)	—
Nursing	218 (61.9)	
Academic year level		
First year	177 (50.3)	—
Second year	175 (49.7)	
Living with older adults (≥ 60)		
Yes	149 (42.3)	—
No	203 (57.7)	
Have you ever studied geriatric physiology?		
Yes	197 (56.0)	—
No	155 (44.0)	
Have you ever studied geriatric psychology?		
Yes	189 (53.7)	—
No	163 (46.3)	
Volunteer service frequency for older adults		
≥ 3	85 (24.1)	—
< 3	267 (75.9)	
FSA	—	64.66 ± 9.45
KAOP	—	143.20 ± 14.17

Abbreviations: FSA, Fraboni scale of ageism; KAOP, Kogan's attitudes toward older people; SD, standard deviation.

### 3.2 Correlation Analysis

A Pearson's linear correlation analysis of the continuous variables is presented in Table 2. There is a significant and negative correlation between FSA and KAOP (*r* = −0.582, *p* < 0.001). This suggests that the more positive the attitude toward the older adults, the lower the degree of ageism.

**TABLE 2:** Pearson's correlation between all continuous variables.

	1	2	3
Age	1		
2 FSA	−0.053	1	
3 KAOP	0.785	−0.582***	1

Abbreviations: FSA, Fraboni scale of ageism; KAOP, Kogan's attitudes toward older people.

*** *p* < 0.001.

### 3.3 Univariate Analysis

In the total sample (*N* = 352), the participants who were willing to pursue a career in OAC accounted for approximately 49.7% (175/352). Table 3 shows the results of univariate analysis. Compared with participants unwilling to pursue a career in OAC, the willing group was significantly more likely to be male (*p* < 0.05), major in aged health care and management (*p* < 0.05), be second‐year students (*p* < 0.05), live with older adults (*p* < 0.05), have participated in volunteer services for the older adults three or more times (*p* < 0.05) and have taken courses in geriatric psychology (*p* < 0.05) and physiology (*p* < 0.05). In addition, the willing group was older (*p* = 0.057), reported lower levels of ageism (FSA) (*p* < 0.05), and held a more positive attitude toward older adults (KAOP) (*p* = 0.055).

**TABLE 3:** Self‐report of career intention in OAC according to characteristics (*n* = 352).

Characteristics	Career intention in OAC		*p*
	Willing (*n* = 175)	Unwilling (*n* = 177)	
Age	19.86 ± 1.07	19.64 ± 1.02	0.057
Gender			
Female	142 (46.9)	161 (53.1)	0.008**
Male	33 (67.3)	16 (32.7)	
Major			
Nursing	81 (37.2)	137 (62.8)	0.000***
Aged health care and management	94 (70.1)	40 (29.9)	
Academic year level			
Second year	101 (57.7)	74 (42.3)	0.003**
First year	74 (41.8)	103 (58.2)	
Living with older adults (≥ 60)			
No	86 (42.4)	117 (57.6)	0.000***
Yes	89 (59.7)	60 (40.3)	
Have you ever studied geriatric physiology?			
No	52 (33.5)	103 (66.5)	0.000***
Yes	123 (62.4)	74 (37.6)	
Have you ever studied geriatric psychology?			
No	55 (33.7)	108 (66.3)	0.000***
Yes	120 (63.5)	69 (36.5)	
Volunteer service frequency for older adults			
< 3	118 (44.2)	149 (55.8)	0.000***
≥ 3	57 (67.1)	28 (32.9)	
FSA	62.90 ± 9.50	66.40 ± 9.09	0.000***
KAOP	144.65 ± 14.40	141.76 ± 13.82	0.055

Abbreviations: FSA, Fraboni scale of ageism; KAOP, Kogan's attitudes toward older people; OAC, older adult care.

** *p* < 0.01.

*** *p* < 0.001.

### 3.4 Score Analysis of FSA

**FIGURE 1:**
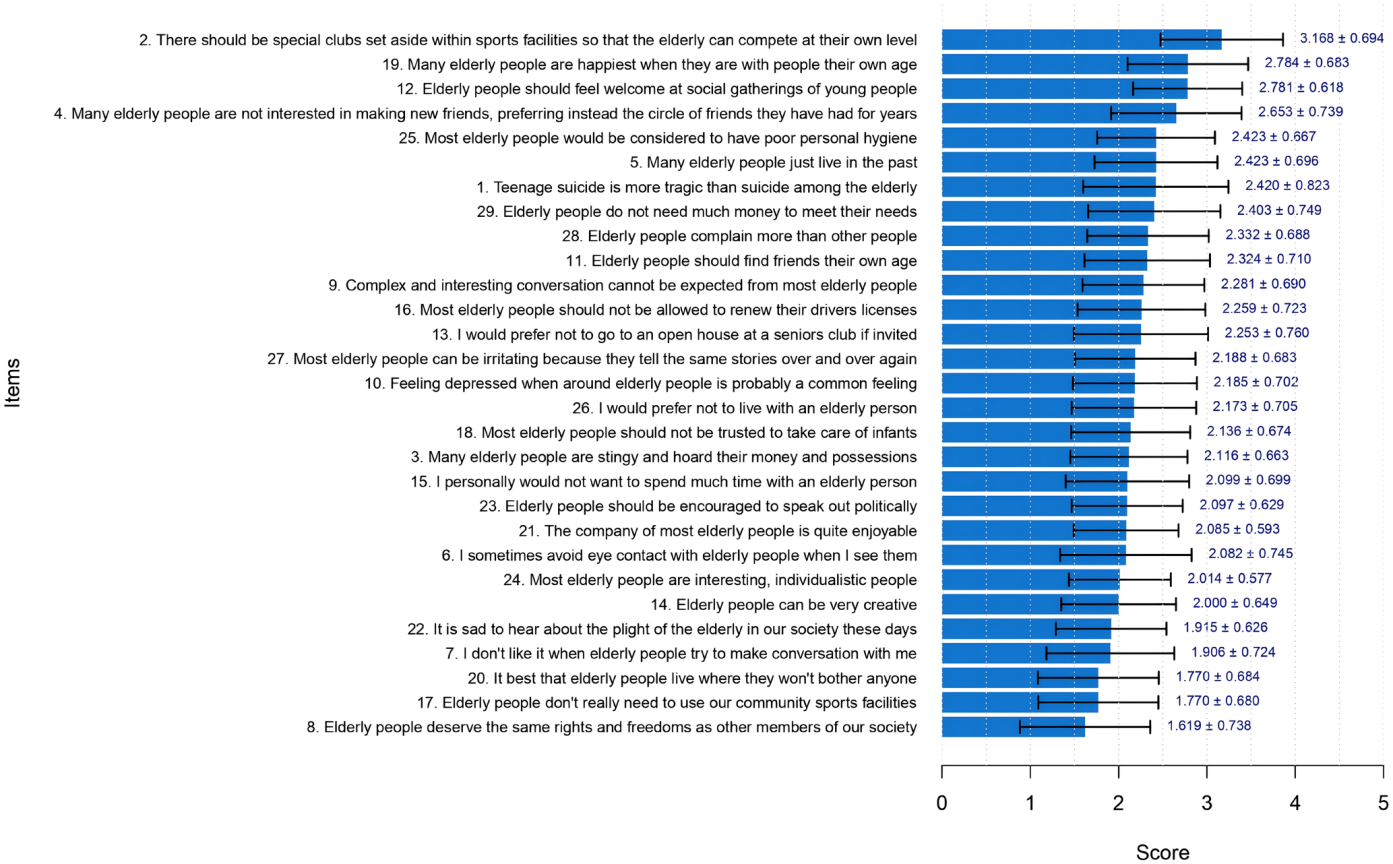
Mean FSA scores for individual questions of the questionnaire. The 8th, 12th, 14th and 21‐24th items were scored in reverse.

Figure 1 presents the mean and standard deviations for each FSA item. Items were ranked by mean score. ‘There should be special clubs set aside within sports facilities so that the elderly can compete at their own level’ had the highest score (3.168 ± 0.694), representing a strong indicator of ageism. This was followed by ‘Many elderly people are happiest when they are with people their own age’ (2.784 ± 0.683) and ‘Elderly people should feel welcome at social gatherings of young people’ (2.781 ± 0.618). Another key indicator was ‘Many elderly people are not interested in making new friends, instead preferring the circle of friends they have had for years’ (2.653 ± 0.739).

### 3.5 Preferred Client Age Groups for Service Provision

**FIGURE 2:**
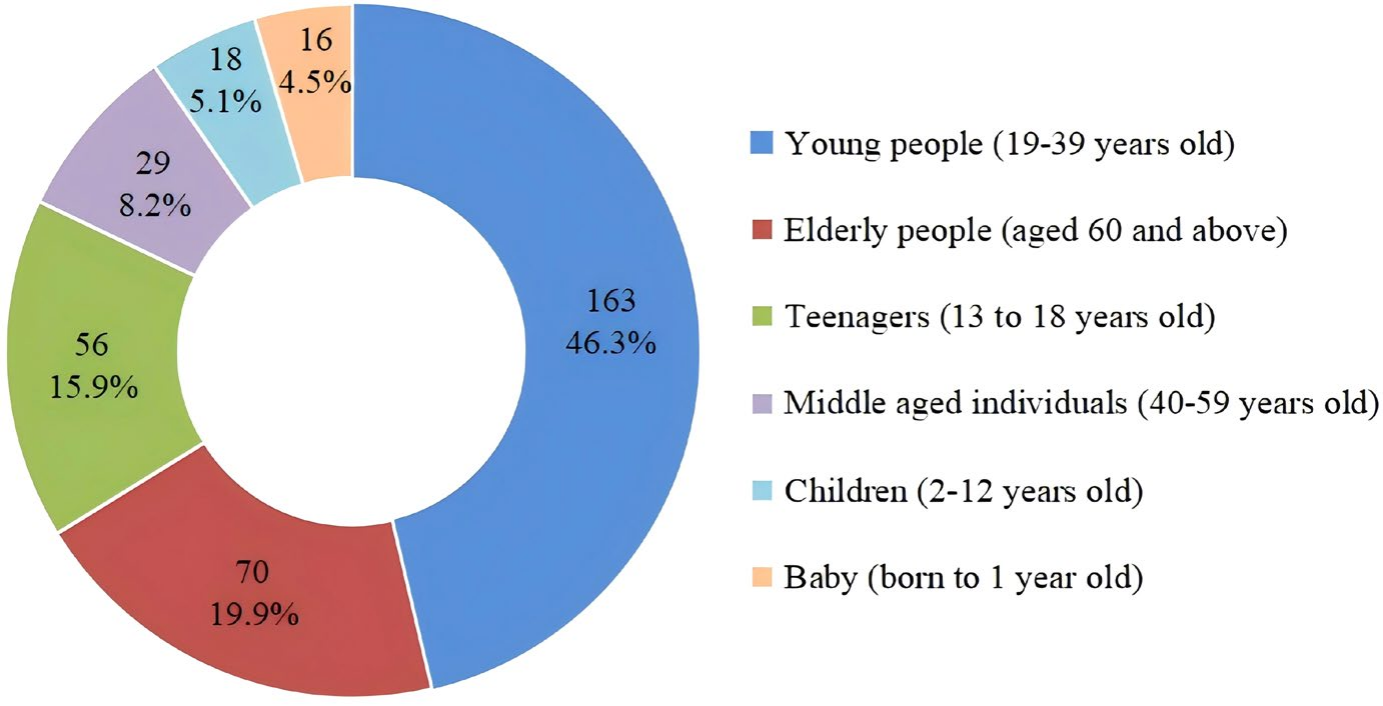
Preferred client age groups for service provision.

Figure 2 shows participants' preferred client age groups for future service provision. Young adults (19–39 years) were the most frequently selected group (46%, 163/352), followed by older adults (≥ 60 years, 20%, 70/352) and adolescents (13–18 years, 16%, 56/352).

### 3.6 Hierarchical Logistic Regression Analysis of Factors Associated With Career Intention

All variables in Table 3 were included in the hierarchical logistic regression. The Hosmer–Lemeshow test indicated good model fit for all three logistic regression models, as all *p* values were > 0.05. Table 4 shows the results of factors related to career intention. All three logistic regression models are significant (*p* < 0.001), the ‘−2 Log likelihood’ of each model (model 1: 422.983, model 2: 421.805, model 3: 418.203, model 4:418.129) and Nagelkerke *R* ^2^ model 1: (0.221, model 2:0.225, model 3: 0.236, model 4: 0.236) were given. Compared with Model 3, Model 4 nearly did not show any changes in the −2 Log likelihood (Δ‐2Log likelihood≈0) and Nagelkerke *R* ^2^ (ΔNagelkerke *R* ^2^ = 0) after adding KAOP, and the regression coefficient of KAOP itself was not significant (*p* > 0.05). This indicates that KAOP fails to provide any additional information independent of FSA and other covariates. Based on the principle of model simplicity, we have chosen Model 2 as the final explanatory model.

**TABLE 4:** Logistic regression analysis of factors associated with career intention.

Variables	Model 1		Model 2		Model 3		Model 4	
	*p*	OR (95% CI)	*p*	OR (95% CI)	*p*	OR (95% CI)	*p*	OR (95% CI)
Age	0.809	0.969 (0.749–1.253)	0.806	0.968 (0.748–1.253)	0.818	0.970 (0.747–1.259)	0.818	0.970 (0.747–1.260)
Gender	0.032	2.187 (1.068–4.479)*	0.033	2.182 (1.065–4.473)*	0.023	2.315 (1.122–4.776)*	0.022	2.334 (1.128–4.831)*
Female								
Male								
Major	0.002	3.142 (1.516–6.511)**	0.002	3.093 (1.489–6.424)**	0.002	3.138 (1.505–6.544)**	0.002	3.153 (1.511–6.579)**
Nursing								
Aged health care and management								
Academic year level	0.545	1.197 (0.669–2.144)	0.575	1.182 (0.659–2.122)	0.560	1.191 (0.662–2.141)	0.552	1.195 (0.664–2.149)
Second year								
First year								
Living with older adult (≥ 60)	0.012	1.829 (1.145–2.924)*	0.016	1.785 (1.114–2.860)*	0.044	1.639 (1.014–2.650)*	0.045	1.636 (1.011–2.645)*
No								
Yes								
Have you ever studied geriatric physiology?	0.516	1.300 (0.590–2.866)	0.524	1.294 (0.586–2.861)	0.453	1.360 (0.609–3.038)	0.446	1.367 (0.611–3.056)
No								
Yes								
Have you ever studied geriatric psychology?	0.538	1.299 (0.565–2.984)	0.540	1.299 (0.563–2.993)	0.710	1.175 (0.502–2.750)	0.724	1.166 (0.497–2.734)
No								
Yes								
Frequency of volunteer services	0.015	2.037 (1.151–3.604)*	0.014	2.044(1.153‐3.623)*	0.024	1.945 (1.092–3.465)*	0.025	1.934 (1.084–3.451)*
< 3								
≥ 3								
FSA		—	0.279	1.009 (0.993–1.026)	0.030	0.972 (0.947–0.997)*	0.056	0.969 (0.939–1.001)
KAOP		—				—	0.786	0.997 (0.977–1.018)
−2 Log likelihood		422.983		421.805		418.203		418.129
Nagelkerke *R* ^2^		0.221		0.225		0.236		0.236

Abbreviations: CI, confidence interval; FSA, Fraboni scale of ageism; KAOP, Kogan's attitudes toward older people; OR, odds ratio.

* *p* < 0.05.

** *p* < 0.01.

The optimal model (Model 3) results show that male gender (OR = 2.135, 95% CI = 1.022–4.776), majoring in aged health care and management (OR = 3.138, 95% CI = 1.505–6.544), living with older adults (OR = 1.639, 95% CI = 1.014–2.650), and higher frequency of volunteering for older adults (≥ 3 times) (OR = 1.945, 95% CI = 1.092–3.465) were positively associated with career intention in OAC. Meanwhile, ageism was negatively associated with the intention to pursue a career in OAC (OR = 0.972, 95% CI = 0.947–0.997).

## 4 Discussion

Approximately half of the participants expressed a willingness to pursue a career in OAC (49.7%, 175/352). The proportion was relatively high among students majoring in aged health care and management (70.1%), whereas it was considerably lower among nursing students (37.2%). Male gender, majoring in aged health care and management, higher frequency of volunteering for older adults (≥ 3 times), living with older adults, and lower level of ageism were positively associated with the intention to pursue a career in OAC. Notably, only 20% of all participants chose older adults as their preferred group to serve in the future. This finding underscores the existing gap between population aging demands and students' actual service preferences, suggesting that targeted interventions are urgently needed to enhance their willingness to serve older adults.

Despite these preferences, nearly all nursing graduates are expected to work with older adults owing to demographic trends, regardless of personal inclination (Nienke 2012). However, OAC remains unpopular among nursing students globally (Koskinen et al. 2022; Dai et al. 2021; Backhaus et al. 2018; Araújo et al. 2023). This reluctance is often attributed to perceptions of OAC as physically demanding, low in status, and poorly compensated (Dai et al. 2021; Shen and Xiao 2012). Consistent with previous studies, most nursing students in this study did not intend to pursue a career in OAC. Encouragingly, evidence suggests that the likelihood of nurses entering OAC seems to increase over time, particularly among those seeking leadership roles or part‐time work (Abrahamsen 2019). In contrast, most aged health care and management students expressed willingness to work in OAC. This disparity reflects differences in career orientation, curriculum, professional perceptions, and job expectations. Unlike nursing education, which focuses on disease‐oriented care and acute settings (Garbrah et al. 2017), the aged health care and management programme was tailored to OAC, emphasizing social value and integrated services. To enhance OAC career willingness, future training should integrate OAC‐oriented curricula and diverse career pathways from aged health care and management programmes into nursing education, combining technical expertise with a broader, more attractive framework.

Most studies indicated that women in geriatric nursing were more positive (Watson et al. 2020), however, this study found that men were more likely than women to express willingness to work in OAC. This shift may reflect the evolving appeal of the OAC sector to students of different genders. First, assisting disabled or semi‐disabled older adults often requires physical strength (Ben Natan et al. 2015). Second, the OAC industry transitions from basic care to more specialized and diversified services; new positions are emerging that focus more on technical skills, management coordination, and cross‐disciplinary collaboration areas that align with some men's career interests. Third, while OAC has traditionally been viewed as flexible work dominated by women, there is growing recognition that it is a professional service rather than a form of domestic labour.

This study also found that students who had volunteered with older adults and who lived with them at home were more willing to pursue a career in OAC. One study in China reported that nursing students participating in volunteer services for older adults gained greater knowledge about aging, improved their attitudes toward this population, and were more willing to pursue OAC careers (Yaofang et al. 2022). Another Chinese study identified participation in older adults related activities as a predictor of willingness to work with older adults (Guo et al. 2021). Such interactions have been shown to reduce students' aging‐related anxiety (Yan et al. 2011). Previous studies have shown that students with experience caring for older adults demonstrate greater confidence and skill in providing care (Zhang et al. 2016), and such experience increases their intention to work in OAC (Garbrah et al. 2017). Given that an increasing number of nuclear families in China no longer live with grandparents, student education should include targeted measures to increase direct interaction with older adults, such as voluntary service and practical care activities, to reduce students' resistance through meaningful contact.

Prejudice against older adults is a key factor hindering students from working with older adults (Shen and Xiao 2012). It will also have a negative impact on the willingness of medical students to pursue geriatric medicine (Zhao et al. 2020; Gherman et al. 2022). Consistent with previous research, this study identified ageism as a significant barrier to pursuing a career in OAC. Therefore, training for future OAC professionals must actively challenge stereotypical views of older adults and reflect their real social roles. Educational institutions should prioritize integrating anti‐ageism content into curricula to dismantle stereotypes, for instance, through case studies on age‐based discrimination and interactive workshops with older adults.

However, no significant correlation was found between KAOP and career intention. Previous studies have found that even students who hold a positive attitude toward older adults often express little interest in pursuing careers in OAC (Dai et al. 2021; Guo et al. 2021). In this study, both univariate and multivariable logistic regression indicated that KAOP did not significantly predict willingness to work in OAC. FSA alone was an important predictor for career intention. However, when KAOP and FSA entered together in Model 4, FSA became non‐significant, while KAOP remained non‐significant. Correlation analysis showed a moderate negative correlation between KAOP and FSA (*r* = −0.582, *p* < 0.001) in this study, which is consistent with the theoretical expectation of inverse positive/negative attitudes toward a group. The VIF values of KAOP and FSA were much less than 5, thus ruling out severe multicollinearity, indicating that the loss of significance of ageism and the persistent non‐significance of KAOP stemmed from intrinsic variable relationships, rather than collinearity‐induced standard error inflation. So a plausible explanation is that KAOP lacks independent predictive efficacy and shares substantial overlapping variance with FSA; they may reflect opposite sides of the same psychological structure (overall attitude toward older adults). When both are included, the non‐informative variance of KAOP weakens the independent predictive effect of FSA (a suppression effect), and KAOP provides no new predictive information beyond what ageism already covers.

### 4.1 Limitations

This study has several limitations. First, the use of a cross‐sectional survey design prevents causal inference. Thus, longitudinal research is needed to explore causal relationships. Second, participants were drawn from only two OAC‐related majors at a single institution. Therefore, the findings may not be generalizable to all vocational education students across China. Third, this study adopted a convenience sampling method, which is commonly used in this type of research but may introduce selection bias. This bias could potentially affect the representativeness of the sample, as convenience sampling may not fully capture the diversity of the target population, and future studies are encouraged to adopt more rigorous sampling methods (e.g., stratified sampling) to mitigate this limitation. Fourth, future studies should consider the inherent correlations and structural overlap when selecting predictors related to attitudes toward older adults, prioritize indicators with stronger explanatory power and greater independence, and avoid including redundant variables that may reduce the explanatory efficacy of core variables, thereby improving model stability and predictive efficiency.

## 5 Conclusion

This study suggests that students' majors, interactions with older adults, and ageism are related to their future career intention in OAC. The identified factors can be used to guide the development of talent cultivation strategies for OAC, in order to promote the development of OAC human resources in China.

## Funding

We would like to express our sincere appreciation for the individuals who aided us during the process of data collection and analysis. This study was funded by the Zhejiang Provincial Philosophy and Social Sciences Planning Project (Grant No. 22NDJC041Z), Ningbo Public Welfare (Key) Science and Technology Plan Project (Grant No. 2023S027), and the Teaching Reform Project of Ningbo College of Health Sciences (Grant No. 2024JY06).

## Ethics Statement

This study has been performed in accordance with the Declaration of Helsinki. All eligible participants signed an informed consent before participating in the study. And ethics approval was obtained from the ethics review committee of Ningbo College of Health Sciences.

## Consent

The authors have nothing to report.

## Conflicts of Interest

The authors declare no conflicts of interest.

## Data Availability

The data in this study cannot be shared.
